# Decreased macrophage inflammatory protein (MIP)-1α and MIP-1β increase the risk of developing nasopharyngeal carcinoma

**DOI:** 10.1186/s40880-018-0279-y

**Published:** 2018-04-03

**Authors:** Meng-Jie Yang, Jie Guo, Yan-Fang Ye, Sui-Hong Chen, Li-Xia Peng, Chu-Yang Lin, Ting Hu, Shang-Hang Xie, Chuan-Bo Xie, Qi-Hong Huang, Yu-Qiang Lu, Qing Liu, Chao-Nan Qian, Su-Mei Cao

**Affiliations:** 1 State Key Laboratory of Oncology in South China, Collaborative Innovation Center for Cancer Medicine, Sun Yat-sen University Cancer Center, Guangzhou, 510060 Guangdong P. R. China; 2 Department of Cancer Prevention Research, Sun Yat-sen University Cancer Center, 651 Dongfeng Road East, Guangzhou, 510060 Guangdong P. R. China; 3 School of Public Health, Sun Yat-sen University, Guangzhou, 510060 Guangdong P. R. China; 4 Sun Yat-sen Memorial Hospital, Sun Yat-sen University, Guangzhou, 510060 Guangdong P. R. China; 5 Department of Experimental Research, Sun Yat-sen University Cancer Center, 651 Dongfeng Road East, Guangzhou, 510060 Guangdong P. R. China; 6 Sihui Cancer Institute, Sihui, 526200 Guangdong P. R. China

**Keywords:** Nasopharyngeal carcinoma, Prospective study, Inflammatory cytokine, Macrophage inflammatory protein, Epstein–Barr virus

## Abstract

**Background:**

The association of circulating inflammation markers with nasopharyngeal carcinoma (NPC) is still largely unclear. This study aimed to comprehensively explore the relationship between circulating cytokine levels and the subsequent risk of NPC with a two-stage epidemiologic study in southern China.

**Methods:**

The serum levels of 33 inflammatory cytokines were first measured in a hospital-based case–control study (150 NPC patients and 150 controls) using multiplex assay platforms. Marker levels were categorized into two or more groups based on the proportion of sample measurements that was above the lower limit of detection. Odds ratios (ORs) and 95% confidence intervals (CIs) relating the serum marker concentration to the risk of NPC were computed by multivariable logistic regression models. The associations were validated in 60 patients with NPC and 120 controls in a subsequent nested case–control study within a NPC screening trial. Potential interactions between serum cytokines and Epstein–Barr virus (EBV) relating to the risk of NPC were assessed using a likelihood ratio test.

**Results:**

The levels of serum macrophage inflammatory protein (MIP)-1α and MIP-1β in the highest categories were associated with a decreased risk of NPC in both the case–control study (MIP-1α: OR = 0.49, 95% CI = 0.26–0.95; MIP-1β: OR = 0.47, 95% CI = 0.22–1.00) and the nested case–control study (MIP-1α: OR = 0.13, 95% CI = 0.03–0.62; MIP-1β: OR = 0.20, 95% CI = 0.04–0.94), compared with those in the lowest categories. Furthermore, individuals with lower levels of these two cytokine markers who were EBV seropositive presented with a largely higher risk of NPC compared with patients with higher levels who were EBV seronegative in both the case–control study (MIP-1α: OR = 16.28, 95% CI = 7.11–37.23; MIP-1β: OR = 12.86, 95% CI = 5.9–28.05) and the nested case–control study (MIP-1α: OR = 86.12, 95% CI = 10.58–701.03; MIP-1β: OR = 115.44, 95% CI = 13.92–957.73).

**Conclusions:**

Decreased preclinical MIP-1α and MIP-1β levels might be associated with a subsequently increased risk of NPC. More mechanistic studies are required to fully understand this finding.

**Electronic supplementary material:**

The online version of this article (10.1186/s40880-018-0279-y) contains supplementary material, which is available to authorized users.

## Background

Nasopharyngeal carcinoma (NPC) shows a distinct difference in geographical distribution globally. Although it is rare in most countries worldwide, with an incidence of less than 1 per 100,000 person-years, it is common in southern China, with an estimated incidence rate of more than 20 per 100,000 person-years [1, 2]. Among the proposed etiological factors for NPC, including genetics, environmental factors, and life style, Epstein–Barr virus (EBV) infection is considered an important risk factor for NPC initiation and progression [3, 4]. EBV DNA is consistently detected in all NPC tissues [5, 6]. Anti-EBV antibody titers can be elevated for several years before clinical evidence of NPC appears, and they have been used for NPC screening in NPC endemic regions [7–9]. Additionally, the circulating EBV DNA load has been used as a prognostic marker for monitoring patients after NPC treatment [10, 11]. However, the fact that approximately 95% of the world’s population sustains an asymptomatic EBV infection with a relatively low NPC incidence suggests the involvement of other synergistic risk factors in the process of carcinogenesis in nasopharyngeal epithelial cells, such as genetic susceptibility, smoking, preserved food consumption, and inflammation [12–14].

NPC is characterized by a heavy infiltration of nonmalignant lymphocytes, suggesting that inflammation might be an important co-factor for this cancer. During inflammation, highly reactive nitrogen and oxygen species can be released from inflammatory cells, resulting in permanent genomic alterations in the nasopharyngeal epithelium. These genetic and epigenetic events may facilitate the infection of nasopharyngeal epithelial cells by EBV [15–17]. Inflammation can also disturb overall immune competence, which could promote latent EBV to enter lytic cycles [18, 19].

Measuring circulating levels of inflammatory markers can help to evaluate the relationship of cancer with chronic inflammation. Previous studies [20–22] of inflammatory markers in NPC have detected a few inflammatory markers, such as interleukin (IL)-6, IL-1, IL-10, tumor necrosis factor (TNF)-alpha, and vascular endothelial growth factor (VEGF). However, all these studies were retrospective and could not infer the state of the circulating cytokines prior to the development of NPC [20–23]. Therefore, comprehensive prospective studies are still needed to clarify the relationship between the immune status of precancerous lesions and NPC.

Our previous studies using multiplex assay platforms, which enable the simultaneous evaluation of many circulating cytokines in small amounts of serum, have shown acceptable performance in liver cancer [24] and colorectal cancer cohorts [25]. Therefore, the present study applied this technology to explore the relationship between circulating cytokine levels and risk of subsequent NPC development using a hospital-based case–control study, followed by a nested case–control design in a population-based screening trial in Sihui, China [26]. We also analyzed whether a combination of serum cytokines and EBV could further increase the risk of NPC.

## Materials and methods

### Study population

#### Case–control study

The serum samples of 150 patients with NPC and 150 healthy controls undergoing routine health examinations were consecutively collected from the serum bank of Sun Yat-sen University Cancer Center (SYSUCC). The patients were selected based on the following criteria: (i) Cantonese NPC patients with histologically proven NPC who had not undergone any treatment; (ii) aged 30–59 years old; (iii) lacked any severe inflammation, immune system disease, or diabetes; and (iv) had serum samples collected before treatment. These Cantonese examinees were frequency matched to patients in the same hospital by gender, age group (≤ 40 and > 40 years), and year of blood collection using a 1:1 ratio. The TNM staging for patients with NPC was defined according to the staging system described in the seventh edition of Union for International Cancer Control (UICC), and NPCs were classified by the World Health Organization (WHO) classification [27]. All diagnoses of NPC were proven by biopsy.

#### Nested case–control study

A nested case–control study was conducted complementary to the case–control study. Subjects in the nested case–control study were recruited within an ongoing cluster-randomized NPC screening trial in Sihui, southern China, from May 30, 2008 [28]. In brief, two EBV-related serologic immunoglobulin A antibodies against capsid antigen (VCA/IgA) and EBV nuclear antigen-1 (EBNA1)/IgA were used as screening markers. The inclusion criteria were: (i) Cantonese patients who were aged 30–59 years, (ii) lack of any recorded history of NPC, and (iii) in good physical condition and mental health. The exclusion criteria were: (i) patients who had severe cardiovascular, liver, or kidney diseases or immune deficiency disease, and (ii) patients with prevalent NPC. Eligible participants were invited to donate 6 mL of blood for serological tests; their basic information was collected. Blood samples were allowed to clot at room temperature, followed by centrifugation at 2000×*g* for 10 min. The serum samples were then aliquoted and stored at − 80 °C. No more than two freeze–thaw cycles were allowed for each serum sample. According to the baseline serological results, the participants were advised to undergo nasopharynx endoscopic examinations or follow-up at different intervals [26, 29]. Controls free of cancer were individually matched at a 2:1 ratio to case patients based on age (varying within 1 year), gender, year of enrollment, and follow-up years. All subjects gave written informed consent, and human subject approval was obtained from the Institutional Review Board of SYSUCC (No. YP2009051). The data from this study have been uploaded onto the Research Data Deposit public platform (http://www.researchdata.org.cn), with the approval RDD number RDDA2017000189.

### Serum cytokine analysis

The serum levels of 33 immune and inflammation markers in 25 µL of baseline serum specimens in the case–control study were measured using the human cytokine/chemokine magnetic bead panel (Millipore, Billerica, MA, USA). The 33 cytokine markers included in this panel are listed in Additional file 1: Table S1. The serum samples were blinded to the measurer and assayed in duplicate, and the average concentrations were calculated for each cytokine. These markers were evaluated for the performance and reproducibility of multiplexed immune/inflammation assays on the basis of a recent methodological study [30]. The concentrations of markers in the serum samples were measured according to the manufacturer’s standard protocol and eventually analyzed using the Luminex 200 analyzer (Luminex, Austin, TX, USA). The concentrations were calculated with a standard curve (ranging from 3.2 to 10,000 pg/mL) made by five-fold dilutions of the human cytokine reconstituted standard in the provided assay buffer. The serum samples were randomly assigned to plates to avoid assay bias. The coefficients of variation (CVs) and intraclass correlation coefficients (ICCs) of quality controls provided by the manufacturer were computed to evaluate the reproducibility of assays.

### Detection of anti-EBV antibodies

The levels of EBNA1/IgA were detected using a commercial enzyme-linked immunosorbent assay (ELISA) kit (Zhongshan Bio-Tech Company, Zhongshan, China) for all samples in this study. The relative optical density (rOD) was calculated by dividing the optical density of one sample by that of a reference control, and the positive criteria was set as 1.5. The ICCs and their 95% confidence intervals (CIs) of quality controls were calculated to ensure the reliability of the serological results.

### Statistical analysis

Samples with values below the assay lower limit of detection were assigned a value of half the lower limit of detection. Marker levels were categorized into groups on the basis of the proportion of samples with measurements above the lower limit of detection as follows [31, 32]: Markers with more than 75% of measurements above the lower limit of detection were categorized into quartiles on the basis of the distribution among controls, markers with 50%–75% of measurements above the lower limit of detection were categorized into three groups (lower than lower limit of detection, lower than median detectable level among controls, or higher than median detectable level), and markers with less than 50% of measurements above the lower limit of detection were categorized into two groups (lower than lower limit of detection and detectable level). Tests for trend were conducted for markers with three or more categories by modeling the intracategory medians as a continuous parameter. Differences in marker levels between patients with NPC and healthy controls were determined using the Wilcoxon rank-sum test. Unconditional logistic regression models were used to compute odds ratios (ORs) and 95% CIs relating the serum marker concentration to the risk of NPC in the case–control study, and conditional logistic regression models were used similarly for the nested case–control study following adjustment for age, gender, salted fish consumption, family history, and EBNA1/IgA. The case patients in the nested case–control were stratified into patients who were diagnosed within 1 year and patients who were diagnosed more than 1 year after the blood was collected. Potential interactions between EBV infection and these immune cytokine markers were assessed using the likelihood ratio test. Correlation between cytokine levels was performed on original serum cytokine levels, using the Spearman correlation coefficient. All tests of statistical significance were two sided at *α* = 0.05. Analyses were performed using SAS version 9.2 (SAS Inc, Cary, NC, USA).

## Results

### Characteristics of participants in the case–control study and the nested case–control study

The distributions of patients and controls by selected demographic and clinicopathologic characteristics are summarized in Table 1. In both case–control studies, patients with NPC and controls had similar gender and age distributions. The average ages of patients with NPC and healthy controls were 45.7 vs. 45.7 years old, respectively, in the case–control study, and 47.8 vs. 47.8 years old, respectively, in nested case–control study.

**Table 1:** Nasopharyngeal carcinoma (NPC) and healthy control patient characteristics in the case–control and nested case–control studies

Characteristic	Case–control study [cases (%)]			Nested case–control study [cases (%)]		
	NPC patients	Controls	*P* value	NPC patients	Controls	*P* value
Total	150	150		60	120	
Gender			1.000			1.000
Male	130 (86.7)	130 (86.7)		35 (58.3)	70 (58.3)	
Female	20 (13.3)	20 (13.3)		25 (41.7)	50 (41.7)	
WHO histological classification						
I	5 (3.3)	NA		2 (3.3)	NA	
II	3 (2.0)	NA		1 (1.7)	NA	
III	142 (94.7)	NA		57 (95.0)	NA	
Clinical stage						
Early (I + II)	51 (34.0)	NA		35 (60.3)	NA	
Advanced (III + IV)	99 (66.0)	NA		23 (39.7)	NA	
Unknown	0	NA		2	NA	
EBNA1/IgA						
Seronegative	52 (34.7)	120 (80.0)	< 0.001	28 (46.7)	115 (95.8)	< 0.001
Seropositive	98 (65.3)	30 (20.0)		32 (53.3)	5 (4.2)	

*NA* not applicable, *NPC* nasopharyngeal carcinoma, *I* squamous cell carcinoma, *II* differentiated nonkeratinizing carcinoma, *III* undifferentiated carcinoma, *Early* clinical stage I and clinical stage II, *Advanced* clinical stage III and clinical stage IV, *EBNA1/IgA* anti-Epstein–Barr virus nuclear antigen-1 immunoglobulin A antibody, *seronegative* the relative optical density (rOD) of the sample was < 1.5, *seropositive* the rOD of the sample was ≥ 1.5

In the nested case–control study, NPC cases and controls were selected from an NPC screening cohort in Sihui. In brief, 11,993 individuals were recruited into the NPC screening program. As of December 31, 2015, 63 NPCs were diagnosed in the screened population. Due to three of these patients lacking baseline blood samples, a total of 60 patients with NPC and 120 controls were included in the nested case–control study.

According to available information on cancer staging, the most (> 95%) pathological tumor type in these two studies was nonkeratinizing carcinoma, and the levels of EBNA1/IgA were clearly higher in patients with NPC than in controls (65.3% vs. 20.0% in the case–control study and 53.3% vs. 4.2% in the nested case–control study). However, the nested case–control study had more early-stage patients (60.3%) than the case–control study (34.0%).

### Measurement qualities of serum cytokines and anti-EBV antibodies

In the case–control study, the 19 cytokine markers with less than 30% of sample measurements above the lower limit of detection were excluded from the analysis. After this exclusion, 14 markers were included in the statistical analysis for each of the two case–control studies. The CVs of the 14 detectable cytokine markers were between 15.6% and 27.9% in the case–control study. For the nested case–control study, the CVs were between 5.1% and 15.5%. The ICCs of the 14 markers included in further analysis for both the case–control study and nested case–control study were all above 0.90 (Additional file 1: Table S1). The ICC of EBNA1/IgA was 0.99 (95% CI 0.91–0.99) in the case–control study and 0.98 (95% CI 0.96–0.99) in the nested case–control study.

### Association of serum cytokines with the risk of NPC

In the case–control study, the median levels of macrophage inflammatory protein (MIP)-1α, MIP-1β, epidermal growth factor (EGF), granulocyte colony-stimulating factor (GCSF), fractalkine, growth-regulated oncogene (GRO), and IL-1α were all significantly lower (*P *< 0.05) in patients with NPCs than in controls. However, only MIP-1β had a similar result (*P *< 0.05) in the nested case–control study. In contrast, the median level of monocyte chemotactic protein 1 (MCP-1) in the case–control study was significantly higher in patients with NPC than in controls (Table 2).

**Table 2:** The levels of 14 markers in nasopharyngeal carcinoma (NPC) patients and control subjects

Marker	Case–control study							Nested case–control study						
	NPC patients (pg/mL)			Controls (pg/mL)			*P*	NPC patients (pg/mL)			Controls (pg/mL)			*P*
	Median	P_25_	P_75_	Median	P_25_	P_75_		Median	P_25_	P_75_	Median	P_25_	P_75_	
EGF	209.3	125.8	327.1	392.5	238.9	594.4	< 0.001	442.3	274.5	583.5	477.0	333.4	629.0	0.26
Eotaxin	126.6	95.4	174.4	143.6	106.2	186.9	0.098	140.0	96.1	195.4	139.1	96.0	212.0	0.86
GCSF	6.5	1.6	15.6	15.6	4.3	50.9	< 0.001	22.8	7.6	40.6	18.6	5.8	31.2	0.13
Fractalkine	11.4	11.4	11.4	59.2	11.4	211.2	< 0.001	92.9	11.4	547.2	92.9	11.4	380.4	0.76
IFN-γ	6.0	1.6	25.5	10.0	3.6	24.5	0.079	5.8	1.6	11.6	4.9	1.6	11.7	0.87
GRO	940.6	731.4	1272.0	1406.0	964.6	2309.5	< 0.001	1926.0	1346.5	4808.5	1615.5	1209.5	2652.5	0.13
MDC	861.3	669.8	1122.0	894.4	590.7	1179.0	0.89	883.3	625.1	1248.5	814.0	628.0	1122.0	0.55
IL-1α	4.7	4.7	4.7	4.7	4.7	68.8	< 0.001	4.7	4.7	24.4	4.7	4.7	98.0	0.08
IL-7	1.6	1.6	7.3	3.2	1.6	11.9	0.11	3.3	1.6	5.3	1.6	1.6	3.7	0.035
IL-8	20.4	7.8	39.8	16.4	7.5	34.0	0.48	58.8	7.3	269.6	108.2	13.3	380.4	0.106
MCP-1	465.2	348.3	598.1	400.0	232.9	568.8	0.004	617.3	400.0	789.0	598.1	450.8	788.8	0.38
MIP-1α	1.6	1.6	66.5	29.0	1.6	95.4	0.023	25.9	1.6	57.0	32.6	4.3	114.5	0.105
MIP-1β	51.5	25.8	82.6	62.6	32.0	102.2	0.036	69.8	31.2	116.7	88.5	43.8	157.8	0.045
VEGF	297.7	129.0	594.4	279.6	129.0	459.3	0.46	218.8	79.0	357.2	188.2	88.5	364.1	0.73

*NPC* nasopharyngeal carcinoma, *P* computed using a Wilcoxon rank-sum test, *EGF* epidermal growth factor, *GCSF* granulocyte colony-stimulating factor, *IFN-γ* interferon gamma, *GRO* growth-regulated oncogene, *MDC* macrophage-derived chemokine, *IL-1α* interleukin-1 alpha, *IL-7* interleukin-7, *IL-8* interleukin-8, *MCP-1* monocyte chemotactic protein-1, *MIP-1α* macrophage inflammatory protein-1 alpha, *MIP-1β* macrophage inflammatory protein-1 beta, *VEGF* vascular endothelial growth factor

The results of multivariable logistic regression analyses between serum cytokine levels and risk of NPC are summarized in Table 3. After adjustment for age, sex, salted fish consumption, family history, and EBNA1/IgA, both MIP-1α and MIP-1β remained significantly different between cases and controls in both studies. The levels of MIP-1α and MIP-1β in the highest category were associated with a statistically significantly decreased risk of NPC compared with those in category 1, both in the case–control study (MIP-1α: OR = 0.49, 95% CI = 0.26–0.95; MIP-1β: OR = 0.47, 95% CI = 0.22–1.00) and in the nested case–control study (MIP-1α: OR = 0.13, 95% CI = 0.03–0.62; MIP-1β: OR = 0.20, 95% CI = 0.04–0.94). In the case–control study, the participants with serum EGF, GCSF, fractalkine, GRO, IL-1α, and IL-7 levels in the highest categories were significantly associated with a decreased risk of NPC compared with those in the lowest categories, whereas MCP-1 was significantly associated with the increased risk of NPC, after adjustment for age, sex, salted fish consumption, family history, and EBNA1/IgA. However, the associations of these markers with the risk of NPC development could not be verified in the nested case–control study.

**Table 3:** Serum cytokine marker associations with nasopharyngeal carcinoma (NPC) in the case–control and nested case–control studies

Marker	Case–control study (cases)^a^					Nested case–control study (cases)				
	NPC patients	Controls	OR_1_ (95% CI)	OR_2_ (95% CI)	OR_3_ (95% CI)	NPC patients	Controls	OR_1_ (95% CI)	OR_2_ (95% CI)	OR_3_ (95% CI)
EGF										
Category 1	84	37	Reference	Reference	Reference	19	30	Reference	Reference	Reference
Category 2	38	37	0.45 (0.25–0.82)	0.40 (0.20–0.78)	0.39 (0.20–0.76)	17	33	0.83 (0.35–1.95)	1.18 (0.31–4.55)	1.17 (0.28–4.85)
Category 3	20	37	0.24 (0.12–0.46)	0.29 (0.14–0.60)	0.30 (0.14–0.62)	11	28	0.63 (0.26–1.54)	0.85 (0.24–2.92)	0.87 (0.22–3.43)
Category 4	8	37	0.10 (0.04–0.22)	0.09 (0.04–0.24)	0.09 (0.04–0.24)	13	29	0.71 (0.29–1.73)	0.46 (0.11–1.90)	0.62 (0.14–2.77)
*P*_trend_			< 0.001	< 0.001	< 0.001			0.383	0.199	0.434
Eotaxin										
Category 1	51	37	Reference	Reference	Reference	15	30	Reference	Reference	Reference
Category 2	37	37	0.73 (0.39–1.35)	0.59 (0.29–1.19)	0.62 (0.30–1.25)	15	31	0.97 (0.43–2.21)	1.08 (0.35–3.30)	1.01 (0.31–3.21)
Category 3	32	37	0.63 (0.33–1.18)	0.60 (0.29–1.22)	0.62 (0.30–1.27)	18	29	1.24 (0.53–2.89)	1.22 (0.36–4.12)	1.05 (0.29–3.84)
Category 4	30	37	0.59 (0.31–1.12)	0.50 (0.24–1.03)	0.53 (0.25–1.10)	12	30	0.80 (0.32–2.01)	1.07 (0.34–3.34)	1.06 (0.30–3.74)
*P*_trend_			0.101	0.072	0.103			0.733	0.894	0.921
GCSF										
Category 1	57	39	Reference	Reference	Reference	14	31	Reference	Reference	Reference
Category 2	52	31	1.15 (0.63–2.10)	1.29 (0.65–2.54)	1.36 (0.68–2.73)	11	33	0.68 (0.26–1.81)	1.05 (0.25–4.42)	1.14 (0.26–5.08)
Category 3	23	35	0.45 (0.23–0.88)	0.39 (0.18–0.84)	0.39 (0.18–0.86)	13	24	1.21 (0.51–2.88)	2.47 (0.70–8.71)	1.91 (0.50–7.38)
Category 4	11	34	0.22 (0.10–0.49)	0.24 (0.10–0.59)	0.24 (0.10–0.58)	21	29	1.57 (0.65–3.80)	2.33 (0.66–8.22)	2.07 (0.53–8.02)
*P*_trend_			< 0.001	< 0.001	< 0.001			0.130	0.104	0.232
Fractalkine										
Category 1	105	60	Reference	Reference	Reference	19	39	Reference	Reference	Reference
Category 2	33	83	0.23 (0.14–0.38)	0.25 (0.14–0.42)	0.25 (0.14–0.45)	13	32	0.79 (0.33–1.91)	1.47 (0.40–5.34)	1.23 (0.40–5.34)
Category 3						19	32	1.44 (0.65–3.17)	2.52 (0.75–8.50)	3.44 (0.49–21.57)
*P*_trend_								0.244	0.145	0.119
IFN-γ										
Category 1	53	33	Reference	Reference	Reference	18	42	Reference	Reference	Reference
Category 2	41	53	0.48 (0.27–0.87)	0.46 (0.23–0.90)	0.47 (0.24–0.92)	21	37	1.34 (0.63–2.86)	1.70 (0.53–5.45)	1.82 (0.52–6.32)
Category 3	46	59	0.49 (0.27–0.87)	0.48 (0.25–0.92)	0.50 (0.26–0.96)	15	37	1.06 (0.46–2.42)	2.31 (0.69–7.68)	2.50 (0.68–9.18)
*P*_trend_			0.092	0.143	0.179			0.966	0.199	0.202
GRO										
Category 1	82	34	Reference	Reference	Reference	8	29	Reference	Reference	Reference
Category 2	44	37	0.49 (0.27–0.89)	0.44 (0.23–0.86)	0.44 (0.23–0.86)	15	31	1.62 (0.61–4.31)	0.90 (0.23–3.48)	0.63 (0.14–2.84)
Category 3	18	31	0.24 (0.12–0.49)	0.25 (0.11–0.54)	0.26 (0.12–0.57)	12	30	1.22 (0.41–3.65)	0.52 (0.12–2.34)	0.36 (0.07–1.76)
Category 4	6	34	0.07 (0.03–0.19)	0.08 (0.03–0.21)	0.08 (0.03–0.21)	18	25	2.40 (0.96–6.04)	1.69 (0.50–5.76)	1.01 (0.26–3.87)
*P*_trend_			< 0.001	< 0.001	< 0.001			0.064	0.229	0.622
MDC										
Category 1	27	38	Reference	Reference	Reference	17	33	Reference	Reference	Reference
Category 2	49	36	1.92 (1.00–3.69)	2.27 (1.08–4.75)	2.21 (1.06–4.63)	6	27	0.46 (0.16–1.33)	0.28 (0.06–1.18)	0.29 (0.07–1.22)
Category 3	43	38	1.59 (0.82–3.08)	1.40 (0.67–2.96)	1.46 (0.69–3.07)	18	31	1.17 (0.51–2.72)	1.15 (0.35–3.76)	1.29 (0.36–4.59)
Category 4	30	37	1.14 (0.57–2.27)	1.22 (0.56–2.65)	1.24 (0.57–2.71)	19	29	1.36 (0.55–3.36)	1.59 (0.45–5.69)	1.34 (0.35–5.06)
*P*_trend_			0.860	0.794	0.902			0.261	0.264	0.448
IL-1α										
Category 1	125	77	Reference	Reference	Reference	40	73	Reference	Reference	Reference
Category 2	12	51	0.15 (0.07–0.29)	0.17 (0.08–0.37)	0.17 (0.08–0.37)	11	36	0.73 (0.34–1.56)	0.97 (0.38–2.48)	1.17 (0.43–3.17)
IL-7										
Category 1	76	69	Reference	Reference	Reference	29	82	Reference	Reference	Reference
Category 2	48	37	1.18 (0.69–2.02)	0.85 (0.46–1.58)	0.85 (0.46–1.60)	26	35	2.01 (1.03–3.93)	1.32 (0.56–3.12)	0.99 (0.39–2.51)
Category 3	19	37	0.47 (0.25–0.89)	0.40 (0.19–0.83)	0.40 (0.19–0.83)					
*P*_trend_			0.018	0.014	0.014					
IL-8										
Category 1	35	35	Reference	Reference	Reference	19	29	Reference	Reference	Reference
Category 2	30	37	0.81 (0.41–1.59)	0.66 (0.31–1.41)	0.68 (0.32–1.45)	20	29	1.05 (0.46–2.42)	1.08 (0.29–3.92)	1.41 (0.36–5.53)
Category 3	38	33	1.15 (0.60–2.23)	0.66 (0.31–1.43)	0.70 (0.32–1.52)	8	27	0.38 (0.13–1.09)	0.18 (0.04–0.96)	0.18 (0.03–1.08)
Category 4	45	34	1.32 (0.69–2.53)	1.05 (0.51–2.18)	1.09 (0.52–2.28)	12	28	0.55 (0.21–1.44)	0.24 (0.04–1.33)	0.26 (0.04–1.83)
*P*_trend_			0.220	0.461	0.412			0.169	0.086	0.167
MCP-1										
Category 1	11	38	Reference	Reference	Reference	22	30	Reference	Reference	Reference
Category 2	45	37	4.20 (1.89–9.35)	2.89 (1.20–6.92)	2.84 (1.19–6.80)	6	29	0.17 (0.04–0.65)	0.28 (0.06–1.35)	0.36 (0.07–1.94)
Category 3	48	39	4.25 (1.92–9.40)	3.45 (1.46–8.18)	3.87 (1.62–9.25)	17	30	0.70 (0.30–1.68)	0.52 (0.15–1.77)	0.56 (0.16–1.99)
Category 4	46	36	4.41 (1.98–9.83)	3.15 (1.32–7.56)	3.41 (1.42–8.19)	13	29	0.61 (0.24–1.53)	0.48 (0.14–1.71)	0.45 (0.12–1.73)
*P*_trend_			0.006	0.043	0.023			0.618	0.375	0.279
MIP-1α										
Category 1	83	66	Reference	Reference	Reference	20	31	Reference	Reference	Reference
Category 2	34	42	0.64 (0.37–1.12)	0.58 (0.31–1.09)	0.60 (0.32–1.14)	14	29	0.81 (0.35–1.89)	0.92 (0.30–2.87)	0.73 (0.25–2.13)
Category 3	33	42	0.63 (0.36–1.09)	0.48 (0.25–0.92)	0.49 (0.26–0.95)	21	30	1.15 (0.47–2.83)	1.33 (0.35–5.05)	1.24 (0.47–3.26)
Category 4						5	30	0.29 (0.10–0.85)	0.16 (0.03–0.98)	0.13 (0.03–0.62)
*P*_trend_			0.132	0.034	0.045			0.020	0.025	0.009
MIP-1β										
Category 1	47	37	Reference	Reference	Reference	21	30	Reference	Reference	Reference
Category 2	43	37	0.92 (0.49–1.69)	0.83 (0.41–1.65)	0.85 (0.42–1.70)	17	30	0.84 (0.38–1.85)	0.67 (0.23–1.96)	0.62 (0.20–1.88)
Category 3	35	37	0.75 (0.40–1.40)	0.69 (0.34–1.40)	0.69 (0.34–1.41)	12	30	0.78 (0.34–1.76)	0.63 (0.20–2.03)	0.55 (0.15–2.02)
Category 4	23	37	0.49 (0.25–0.96)	0.45 (0.21–0.97)	0.47 (0.22–1.00)	10	29	0.39 (0.16–0.99)	0.23 (0.06–0.91)	0.20 (0.04–0.94)
*P*_trend_			0.029	0.034	0.041			0.048	0.037	0.043
VEGF										
Category 1	35	39	Reference	Reference	Reference	15	31	Reference	Reference	Reference
Category 2	34	34	1.11 (0.58–2.15)	0.88 (0.41–1.87)	0.81 (0.38–1.74)	8	29	0.62 (0.22–1.75)	0.46 (0.10–2.08)	0.42 (0.09–1.99)
Category 3	27	36	0.84 (0.43–1.64)	0.85 (0.40–1.83)	0.84 (0.40–1.81)	23	30	1.61 (0.65–3.96)	0.73 (0.18–2.98)	0.58 (0.13–2.61)
Category 4	44	36	1.36 (0.72–2.57)	1.25 (0.61–2.57)	1.26 (0.61–2.59)	13	29	0.97 (0.37–2.51)	0.78 (0.24–2.57)	0.67 (0.18–2.48)
*P*_trend_			0.366	0.433	0.391			0.821	0.926	0.784

*NPC* nasopharyngeal carcinoma, *OR*_*1*_ odds ratio computed using logistic regression adjusted for gender and age, *OR*_*2*_ computed using logistic regression adjusted for gender, age, and anti-Epstein–Barr virus nuclear antigen-1 immunoglobulin A antibody (EBNA1/IgA), *OR*_*3*_ computed using logistic regression adjusted for gender, age, salted fish consumption, family history, and EBNA1/IgA, *CI* confidence interval, *Category 1* lower than the lower limit of detection among controls with less than three groups or the first quartile with four groups, *Category 2* detectable level with two groups, lower than median detectable level among controls with three groups, or in the second quartile with four groups, *Category 3* higher than the median detectable level with three groups or in the third quartile with four groups, *Category 4* in the fourth quartile among controls, *P*_*trend*_ computed using Wald statistic of regression model parameters for marker intracategory medians modeled as a continuous variable, *EGF* epidermal growth factor, *GCSF* granulocyte colony-stimulating factor, *IFN-γ* interferon gamma, *GRO* growth-regulated oncogene, *MDC* macrophage-derived chemokine, *IL-1α* interleukin-1 alpha, *IL-7* interleukin-7, *IL-8* interleukin-8, *MCP-1* monocyte chemotactic protein-1, *MIP-1α* macrophage inflammatory protein-1 alpha, *MIP-1β* macrophage inflammatory protein-1 beta, *VEGF* vascular endothelial growth factor

^a^Samples were excluded from the analysis process for the corresponding marker if the marker serum level of each sample exceeded three times the quartile range above the median

### Associations between serum MIP-1α and MIP-1β and the subsequent diagnosis of NPC in the nested case–control study

To remove the potential confounding factor of subclinical malignancies that might change the circulating MIP-1α and MIP-1β levels, we excluded NPC cases diagnosed within 1 year after baseline blood collection in the nested case–control study. In the subset of case patients who were diagnosed more than 1 year after blood collection, the levels of MIP-1α and MIP-1β in category 4 were still statistically significantly associated with a decreased risk of NPC compared with those in the reference category after adjustment for age, sex, salted fish consumption, family history, and EBNA1/IgA (MIP-1α: OR = 0.09, 95% CI = 0.01–0.82, *P*_trend_ = 0.036; MIP-1β: OR = 0.12, 95% CI = 0.01–0.94, *P*_trend_ = 0.047) (Table 4).

**Table 4:** Associations of macrophage inflammatory protein (MIP)-1α and MIP-1β serum levels with nasopharyngeal carcinoma (NPC) risk in the nested case–control study by the time interval between blood collection and diagnosis

Marker^a^	Cases diagnosed within 1 year after blood collection					Cases diagnosed more than 1 year after blood collection				
	NPCs	Controls	OR_1_ (95% CI)	OR_2_ (95% CI)	OR_3_ (95% CI)	NPCs	Controls	OR_1_ (95% CI)	OR_2_ (95% CI)	OR_3_ (95% CI)
MIP-1α										
Category 1	8	14	Reference	Reference	Reference	12	17	Reference	Reference	Reference
Category 2	4	9	0.70 (0.18–2.81)	2.68 (0.20–35.6)	1.03 (0.14–7.60)	10	20	0.81 (0.28–2.36)	0.68 (0.18–2.61)	0.59 (0.15–2.37)
Category 3	9	15	0.94 (0.21–4.18)	10.14 (0.58–177)	1.76 (0.33–9.51)	12	15	1.13 (0.36–3.54)	0.62 (0.12–3.29)	1.21 (0.31–4.70)
Category 4	2	10	0.32 (0.05–1.86)	0.80 (0.06–10.2)	0.10 (0.01–2.39)	2	20	0.16 (0.03–0.81)	0.07 (0.01–0.82)	0.09 (0.01–0.82)
*P*_trend_			0.462	0.450	0.312			0.020	0.032	0.036
MIP-1β										
Category 1	6	13	Reference	Reference	Reference	15	17	Reference	Reference	Reference
Category 2	7	13	1.16 (0.31–4.34)	1.28 (0.20–8.42)	1.79 (0.22–14.7)	10	17	0.72 (0.26–2.00)	0.41 (0.10–1.74)	0.38 (0.09–1.59)
Category 3	7	14	1.08 (0.31–3.83)	1.23 (0.22–6.78)	1.62 (0.20–13.3)	7	14	0.64 (0.21–1.94)	0.32 (0.05–1.95)	0.30 (0.05–1.84)
Category 4	4	8	1.09 (0.26–4.55)	0.83 (0.12–5.72)	0.96 (0.05–17.3)	4	23	0.21 (0.06–0.74)	0.08 (0.01–0.59)	0.12 (0.01–0.94)
*P*_trend_			0.953	0.860	0.919			0.013	0.016	0.047

*MIP-1α* macrophage inflammatory protein-1 alpha, *MIP-1β* macrophage inflammatory protein-1 beta, *NPC* nasopharyngeal carcinoma, *OR*_*1*_ odds ratio computed using logistic regression adjusted for gender and age, *OR*_*2*_ computed using logistic regression adjusted for gender, age, and anti-Epstein-Barr virus nuclear antigen-1 immunoglobulin A antibody (EBNA1/IgA), *OR*_*3*_ computed using logistic regression adjusted for gender, age, salted fish consumption, family history, and EBNA1/IgA, *CI* confidence interval, *Category 1, 2, 3, and 4* markers were categorized into four groups based on the quartiles among controls, *P*_*trend*_ computed using Wald statistic of regression model parameters for marker intracategory medians modeled as a continuous variable

^a^Samples were excluded from the analysis process for the corresponding marker if the marker serum level of each sample exceeded three times the quartile range above the median

### Interaction analysis of MIP-1α, MIP-1β, and EBV status with the risk of NPC

**Fig. 1:**
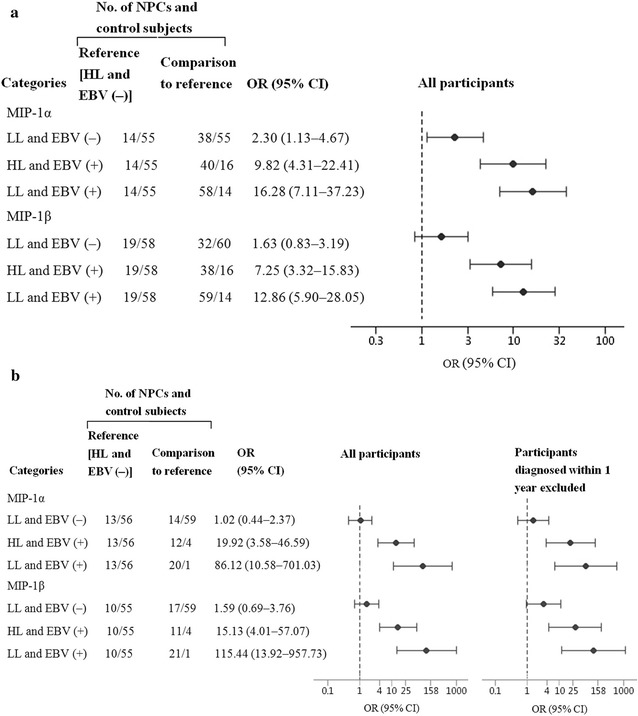
Association of serum macrophage inflammatory protein (MIP)-1α and MIP-1β levels and Epstein–Barr virus (EBV) status with the risk of nasopharyngeal carcinoma (NPC). **a** The case–control study; **b** the nested case–control study. High levels (HL) and low levels (LL) of MIP-1α and MIP-1β in patients with NPC were classified based on the median value among control subjects; EBV(−), seronegative for EBV nuclear antigen-1 immunoglobulin A (EBNA1/IgA); EBV(+), seropositive for EBNA1/IgA. Odds ratios (ORs) and 95% confidence intervals (CIs) were computed using conditional logistic regression adjusted for gender and age. Vertical dashed lines represent an OR of 1.0. Solid black circles represent the ORs, and solid horizontal bars represent the 95% CIs

As EBV infection may influence the level of cytokines in the body, an interaction analysis was further conducted between MIP-1α, MIP-1β, and EBNA1/IgA. MIP-1α and MIP-1β levels were divided into high and low groups based on the median values of control groups, and the EBV status was divided to seronegative [EBV(−)] or seropositive [EBV(+)] according to the presence of EBNA1/IgA. The results show that, in the nested case–control study, the OR increased significantly among EBV(+) participants with low MIP-1α (OR = 86.12, 95% CI = 10.58–701.03) or MIP-1β (OR = 115.44, 95% CI = 13.92–957.73) levels, but no statistically significant interaction between these levels was observed (*P* values of 0.134 and 0.211, respectively). Further, the associations remained statistically significant among case patients who were diagnosed more than 1 year after blood collection (MIP-1α: OR = 66, 95% CI 7.19–606.29; MIP-1β: OR = 113.75, 95% CI = 12.61–1114) (Fig. 1). In the case–control study, low MIP-1α and MIP-1β serum levels in EBV seropositive individuals also showed a higher risk of NPC development (MIP-1α: OR = 16.28, 95% CI = 7.11–37.23; MIP-1β: OR = 12.86, 95% CI = 5.9–28.05).

### Correlations between serum levels of MIP-1α, MIP-1β, and other cytokines in the controls of the case–control study and the nested case–control study

**Fig. 2:**
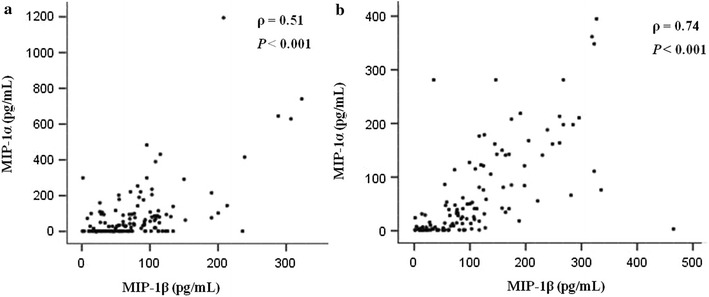
Macrophage inflammatory protein (MIP)-1α and MIP-1β serum level correlations among control subjects in case–control and nested case–control studies. **a** Hospital-based case–control study; **b** nested case–control study based on the NPC Screening Trial in Sihui. *MIP* macrophage inflammatory protein, *ρ* Spearman’s correlation coefficient

The correlations between serum levels of MIP-1α, MIP-1β, and other cytokines were explored among the control subjects in the case–control study and nested case–control study. We found a positive correlation between MIP-1α and MIP-1β levels in both studies (Spearman’s correlation coefficient [ρ] = 0.51, *P *< 0.001 in the case–control study; ρ = 0.74, *P *< 0.001 in the nested case–control study) (Fig. 2). In addition, there were also statistically significant correlations between IL-8, eotaxin, and MCP-1 levels with the levels of these two markers among control subjects both in the case–control study and in the nested case–control study, with ρ values ranging from 0.18 to 0.63 (all *P *< 0.05) (Table 5).

**Table 5:** The relationship between macrophage inflammatory protein (MIP)-1α or MIP-1β and other cytokines

Marker	MIP-1α				MIP-1β			
	Case–control study		Nested case–control study		Case–control study		Nested case–control study	
	ρ	*P*	ρ	*P*	ρ	*P*	ρ	*P*
EGF	0.29	0.070	0.07	0.447	0.25	0.003	0.02	0.798
Eotaxin	0.29	< 0.001	0.25	0.007	0.24	0.003	0.22	0.018
GCSF	0.39	< 0.001	0.06	0.544	0.39	< 0.001	− 0.06	0.544
Fractalkine	0.24	0.004	0.05	0.636	0.37	< 0.001	0.15	0.134
IFN-γ	0.38	0.008	0.09	0.316	0.22	0.008	0.11	0.243
GRO	0.21	0.012	0.06	0.523	0.26	0.002	0.06	0.523
MDC	0.33	< 0.001	0.01	0.922	0.26	0.002	0.03	0.778
IL-1α	0.16	0.07	− 0.01	0.888	0.19	0.03	− 0.08	0.394
IL-7	0.27	0.001	0.08	0.411	0.30	< 0.001	− 0.015	0.875
IL-8	0.47	< 0.001	0.63	< 0.001	0.30	< 0.001	0.49	< 0.001
MCP-1	0.20	0.014	0.19	0.035	0.09	0.280	0.18	0.048
VEGF	0.20	0.017	0.09	0.348	0.37	< 0.001	0.13	0.165

*ρ* Spearman’s correlation coefficient, *MIP-1α* macrophage inflammatory protein-1 alpha, *MIP-1β* macrophage inflammatory protein-1 beta, *EGF* epidermal growth factor, *GCSF* granulocyte colony-stimulating factor, *IFN-γ* interferon gamma, *GRO* growth-regulated oncogene, *MDC* macrophage-derived chemokine, *IL-1α* interleukin-1 alpha, *IL-7* interleukin-7, *IL-8* interleukin-8, *MCP-1* monocyte chemotactic protein-1, *VEGF* vascular endothelial growth factor

## Discussion

Here, we examined the relationship between circulating inflammatory cytokine markers and NPC using a multiplexed immunobead platform in two independent studies. Our results reveal the potential association of decreased MIP-1α and MIP-1β levels with the prospective risk of NPC development in southern China. The association was independent of EBV infection, and the risk increased remarkably with decreasing MIP-1α and MIP-1β levels. Moreover, this relationship remained in the subset of serum samples that were collected more than 1 year before NPC onset. The present findings are also consistent with the previously reported evidence that MIP-1α and MIP-1β levels are inversely related to head and neck squamous cell cancers [33].

MIP-1α and MIP-1β are two important and closely related members of the MIP-1 CC chemokine subfamily. They are produced by a variety of lymphocytes, including monocytes, macrophages, activated T cells, and B cells [34]. Additionally, these chemokines play major roles in the recruitment of leukocytes to infection sites [35], the delivery of interferon (IFN) to mediate protective responses against several kinds of virus infections [36, 37], and the induction of antitumor responses [35]. It was reported that MIP-1β can also inhibit the entry and replication of viruses through chemokine receptor binding [34]. Therefore, we speculate that the mechanisms underlying the link between decreased MIP-1α and MIP-1β levels and the risk of developing NPC are related to the dysfunction of anti-EBV immunity and anti-tumor immunity.

Normally, EBV is in a latent status in B lymphocytes under strict monitoring by the immune system, although the virus may be periodically reactivated in response to environmental stress, such as smoking [38] or the consumption of Cantonese-style salted fish [39] or Chinese herbs [40]. With the defective immunity induced by relatively low MIP-1α or MIP-1β levels, lymphocytes may not move into tissues with EBV replication and become appropriately activated, thus promoting more EBV shedding in the nasopharynx and inducing oncogenic transformation of the infected nasopharyngeal epithelium. As EBV infection is an important risk factor for NPC and can lead to chronic inflammation, it is possible that MIP-1α or MIP-1β may interact with EBV to increase the risk of NPC. We conducted a stratification analysis to assess this possibility, and the results revealed that the OR for NPC development increased prominently in subjects with EBV seropositivity who had a low level of MIP-1α or MIP-1β; however, no significant interaction was found, likely due to the relatively small number of samples in this study.

Statistically significant associations were observed between NPC and the levels of several inflammatory cytokines, i.e., EGF, GCSF, fractalkine, GRO, IL-1α, IL-7, and MCP-1, in the case–control study. These findings for EGF, GCSF, GRO, and MCP-1 are consistent with those from previous case–control studies of other cancers, such as gastric cancer [41, 42], breast cancer [43, 44], and renal cell carcinoma [45]. The associations of these inflammatory cytokines with cancer suggest that changes to a cluster of inflammatory cytokines may be caused by the tumor and could be related to patients’ progress. In contrast to our results, one paper reported that NPC patients in Sichuan, China, a non-NPC endemic area, had elevated IL-1α levels in circulation [46]. The different characteristics of the participants in these two studies, including the different race, pathological types, and EBV infection status, might have affected the IL-1α levels. Thus, additional studies need to be conducted to verify the relationship between IL-1α and NPC in endemic areas.

Several inflammation markers associated with NPC that were reported by other studies were not found in the present study, either because they were not contained in the cytokine panel (such as C-reactive protein) or because they had a lower sensitivity in this platform (such as IL-6 and IL-10) [47, 48]. The main reason for the relatively low detection sensitivity for some markers could be interference from other markers in the platform. Another study using the same Millipore 39-plex panel also showed that 17 markers (43%) had low detection sensitivity [30].

The present study has several strengths. The association of the maximum number of immune markers with the risk of NPC was evaluated in this study. The prospective design of this study minimized the potential bias as a result of disease-induced effects. Moreover, the high validity, reproducibility, and stability of the cytokine testing using a multiplex immunobead platform attributed to good assay performances; specifically, the ICCs of cytokines were all > 0.9, and the CVs of the detectable cytokine markers were all < 0.28.

The study also had some limitations. The immune mechanism disturbance with decreased circulating MIP-1α and MIP-1β levels in the preclinical NPC patients may not reflect the levels of these cytokines in the inflammation lesion site that are directly relevant to NPC development (e.g., EBV-mediated inflammation or nasopharyngeal mucosa-associated inflammation). The relatively small sample size in our study restricted its detection of significant associations between the two markers and NPC development when a Bonferroni correction was used to adjust the probability values (*P*_*ad*_ = *P*/N = 0.05/14 = 0.0036). However, it is unlikely that the MIP-1α and MIP-1β levels decreased in NPC by chance, given that they remained significantly associated with NPC in an independent nested case–control study (Additional file 1: Table S1).

## Conclusions

In conclusion, this study found that serum MIP-1α and MIP-1β levels were inversely correlated with the risk of NPC, suggesting an etiologic role for defective antivirus and antitumor immunity in NPC carcinogenesis. Additional investigations are needed to elucidate the biologic mechanisms underlying this association.

## Additional file

**Additional file 1: Table S1.** Performance characteristics of measurement for 33 inflammation markers.

## Abbreviations

NPC: nasopharyngeal carcinoma
OR: odds ratio
CI: confidence interval
EBV: Epstein–Barr virus
EGF: epidermal growth factor
GCSF: granulocyte colony-stimulating factor
IFN-γ: interferon gamma
GRO: growth-regulated oncogene
MDC: macrophage-derived chemokine
IL-1α: interleukin-1 alpha
IL-7: interleukin-7
IL-8: interleukin-8
MCP-1: monocyte chemotactic protein-1
MIP-1α: macrophage inflammatory protein-1 alpha
MIP-1β: macrophage inflammatory protein-1 beta
VEGF: vascular endothelial growth factor
CV: coefficients of variation
ICC: intra-class correlation coefficient
