# Supplementary material for: Decreased macrophage inflammatory protein (MIP)-1α and MIP-1β increase the risk of developing nasopharyngeal carcinoma
Source: Cancer Commun (Lond). 2018 Apr 3;38:7. doi: 10.1186/s40880-018-0279-y (PMC5993139; doi:10.1186/s40880-018-0279-y)
Supplement: Supplementary file 1 — Additional file 1: Table S1. Performance characteristics of measurement for 33 inflammation markers. [file 40880_2018_279_MOESM1_ESM.docx]

Table S1. Performance characteristics of measurement for 33 inflammation markers

|  | **Case-control study** | | | | | | | **Nested case-control study** | | | | | | |
| --- | --- | --- | --- | --- | --- | --- | --- | --- | --- | --- | --- | --- | --- | --- |
| **marker** | **Percent Detectability** | **CV%** | **95% CI** | | **ICC** | **95% CI** | | **Percent Detectability** | **CV%** | **95% CI** | | **ICC** | **95% CI** | |
| EGF | **99.0** | 15.6 | 10.3 | 31.8 | 0.99 | 0.99 | 1.00 | 100.0 | 11.3 | 5.9 | 70.7 | 0.99 | 0.96 | 1.00 |
| TGF-a | **26.9** | ~ | ~ | ~ | ~ | ~ | ~ | ~ | ~ | ~ | ~ | ~ | ~ | ~ |
| EOTAXIN | **99.0** | 20 | 13.3 | 40.9 | 0.99 | 0.97 | 1.00 | 99.4 | 10.4 | 5.4 | 65.6 | 1.00 | 0.98 | 1.00 |
| GCSF | **74.3** | 20.2 | 13.4 | 41.1 | 0.99 | 0.98 | 1.00 | 81.7 | 8.1 | 4.2 | 51.2 | 0.98 | 0.83 | 1.00 |
| FLT-3L | **21.1** | ~ | ~ | ~ | ~ | ~ | ~ | ~ | ~ | ~ | ~ | ~ | ~ | ~ |
| Fractalkine | **50.7** | 27.9 | 18.4 | 56.8 | 0.99 | 0.96 | 1.00 | 67.8 | 6.8 | 3.5 | 42.6 | 0.98 | 0.88 | 1.00 |
| IFNa-2 | **16.7** | ~ | ~ | ~ | ~ | ~ | ~ | ~ | ~ | ~ | ~ | ~ | ~ | ~ |
| IFN-γ | **71.3** | 22.3 | 14.8 | 45.5 | 0.99 | 0.97 | 1.00 | 66.7 | 14.2 | 7.4 | 89.3 | 1.00 | 0.97 | 1.00 |
| IL-10 | **11.5** | ~ | ~ | ~ | ~ | ~ | ~ | ~ | ~ | ~ | ~ | ~ | ~ | ~ |
| GRO | **100** | 22.2 | 14.6 | 45.1 | 0.99 | 0.99 | 1.00 | 100.0 | 15.5 | 8.1 | 97.6 | 1.00 | 0.97 | 1.00 |
| MCP-3 | **26.7** | ~ | ~ | ~ | ~ | ~ | ~ | ~ | ~ | ~ | ~ | ~ | ~ | ~ |
| MDC | **100** | 19 | 15.6 | 38.8 | 0.99 | 0.99 | 1.00 | 100.0 | 6.4 | 3.3 | 40 | 0.99 | 0.96 | 1.00 |
| IL12-P70 | **16.1** | ~ | ~ | ~ | ~ | ~ | ~ | ~ | ~ | ~ | ~ | ~ | ~ | ~ |
| IL-13 | **2.6** | ~ | ~ | ~ | ~ | ~ | ~ | ~ | ~ | ~ | ~ | ~ | ~ | ~ |
| IL-15 | **1.3** | ~ | ~ | ~ | ~ | ~ | ~ | ~ | ~ | ~ | ~ | ~ | ~ | ~ |
| sCD40L | **2.6** | ~ | ~ | ~ | ~ | ~ | ~ | ~ | ~ | ~ | ~ | ~ | ~ | ~ |
| IL-17α | **29.4** | ~ | ~ | ~ | ~ | ~ | ~ | ~ | ~ | ~ | ~ | ~ | ~ | ~ |
| IL1α | **34.3** | 24.9 | 16.5 | 50.7 | 0.98 | 0.96 | 0.99 | 37.2 | 12.8 | 6.7 | 80.7 | 0.98 | 0.84 | 1.00 |
| IL-9 | **1.3** | ~ | ~ | ~ | ~ | ~ | ~ | ~ | ~ | ~ | ~ | ~ | ~ | ~ |
| IL-1β | **0.0** | ~ | ~ | ~ | ~ | ~ | ~ | ~ | ~ | ~ | ~ | ~ | ~ | ~ |
| IL-2 | **0.0** | ~ | ~ | ~ | ~ | ~ | ~ | ~ | ~ | ~ | ~ | ~ | ~ | ~ |
| IL-3 | **0.0** | ~ | ~ | ~ | ~ | ~ | ~ | ~ | ~ | ~ | ~ | ~ | ~ | ~ |
| IL-4 | **2.6** | ~ | ~ | ~ | ~ | ~ | ~ | ~ | ~ | ~ | ~ | ~ | ~ | ~ |
| IL-5 | **9.0** | ~ | ~ | ~ | ~ | ~ | ~ | ~ | ~ | ~ | ~ | ~ | ~ | ~ |
| IL-6 | **28.3** | ~ | ~ | ~ | ~ | ~ | ~ | ~ | ~ | ~ | ~ | ~ | ~ | ~ |
| IL-7 | **51.7** | 18.9 | 12.5 | 38.4 | 0.99 | 0.98 | 1.00 | 38.3 | 5.1 | 2.7 | 32.1 | 1.00 | 0.99 | 1.00 |
| IL-8 | **92.7** | 19.4 | 12.8 | 39.5 | 0.99 | 0.98 | 1.00 | 94.4 | 9.3 | 4.8 | 58.2 | 0.98 | 0.89 | 1.00 |
| MCP-1 | **100** | 16.7 | 11.1 | 34.1 | 1.00 | 0.99 | 1.00 | 99.4 | 10.2 | 5.3 | 64.2 | 0.99 | 0.94 | 1.00 |
| MIP-1α | **50.3** | 15.7 | 10.3 | 31.9 | 0.94 | 0.85 | 0.98 | 76.7 | 10.6 | 5.6 | 67 | 0.99 | 0.94 | 1.00 |
| MIP-1β | **97** | 16.6 | 11 | 33.9 | 1.00 | 0.99 | 1.00 | 95.6 | 11.3 | 5.8 | 70.7 | 1.00 | 0.99 | 1.00 |
| TNF-α | **24.3** | ~ | ~ | ~ | ~ | ~ | ~ | ~ | ~ | ~ | ~ | ~ | ~ | ~ |
| TNF-β | **1.3** | ~ | ~ | ~ | ~ | ~ | ~ | ~ | ~ | ~ | ~ | ~ | ~ | ~ |
| VEGF | **91.7** | 21.5 | 14.2 | 43.8 | 0.99 | 0.99 | 1.00 | 91.6 | 13.2 | 6.9 | 83.1 | 0.99 | 0.96 | 1.00 |

CV: coefficients of variation; ICC: intra-class correlation coefficient; CI: confidence interval; EGF: epidermal growth factor; FGF-2: fibroblast growth factor 2; TGF-α: transforming growth factor alpha; GCSF: granulocyte colony-stimulating factor; FIT-3L: Fms-related tyrosine kinase 3 ligand; GM-CSF: granulocyte-macrophage colony-stimulating factor; IFNα-2: interferon alpha-2; IFN-γ: interferon gamma; GRO: growth-regulated oncogene; IL-10: interleukin-10; MCP-3: monocyte chemotactic protein-3; IL-12P40: interleukin-12 protein 40; MDC: macrophage-derived chemokine; IL-12P70: interleukin-12 protein 70; IL-13: interleukin-13; IL-15: interleukin-15; sCD40L: soluble CD40 ligand; IL-17α: interleukin-17 alpha; IL-1Rα: interleukin-1 receptor alpha; IL-1α: interleukin-1 alpha; IL-9: interleukin-9; IL-1β: interleukin-1 beta; IL-2: interleukin-2; IL-3: interleukin-3; IL-4: interleukin-4; IL-5: interleukin-5; IL-6: interleukin-6; IL-7: interleukin-7; IL-8: interleukin-8; IP-10: interferon gamma-induced protein 10; MCP-1: monocyte chemotactic protein-1; MIP-1α: macrophage inflammatory protein-1 alpha; MIP-1β: macrophage inflammatory protein-1 beta; TNF-α: tumor necrosis factor alpha; TNF-β: tumor necrosis factor beta; VEGF: vascular endothelial growth factor.
